# Research on the Application of Multi-Source Information Fusion in Multiple Gait Pattern Transition Recognition

**DOI:** 10.3390/s22218551

**Published:** 2022-11-06

**Authors:** Chaoyue Guo, Qiuzhi Song, Yali Liu

**Affiliations:** 1 Department of Mechanical and Engineering, Beijing Institute of Technology, 5 South Zhongguancun Street, Haidian District, Beijing 100081, China; 2 Institute of Advanced Technology, Beijing Institute of Technology, Beijing 100081, China

**Keywords:** exoskeleton, gait pattern transition recognition, multi-source information fusion, multi-classifier fusion

## Abstract

Multi-source information fusion technology is a kind of information processing technology which comprehensively processes and utilizes multi-source uncertain information. It is an effective scheme to solve complex pattern recognition and improve classification performance. This study aims to improve the accuracy and robustness of exoskeleton gait pattern transition recognition in complex environments. Based on the theory of multi-source information fusion, this paper explored a multi-source information fusion model for exoskeleton gait pattern transition recognition in terms of two aspects of multi-source information fusion strategy and multi-classifier fusion. For eight common gait pattern transitions (between level and stair walking and between level and ramp walking), we proposed a hybrid fusion strategy of multi-source information at the feature level and decision level. We first selected an optimal feature subset through correlation feature extraction and feature selection algorithm, followed by the feature fusion through the classifier. We then studied the construction of a multi-classifier fusion model with a focus on the selection of base classifier and multi-classifier fusion algorithm. By analyzing the classification performance and robustness of the multi-classifier fusion model integrating multiple classifier combinations with a number of multi-classifier fusion algorithms, we finally constructed a multi-classifier fusion model based on D-S evidence theory and the combination of three SVM classifiers with different kernel functions (linear, RBF, polynomial). Such multi-source information fusion model improved the anti-interference and fault tolerance of the model through the hybrid fusion strategy of feature level and decision level and had higher accuracy and robustness in the gait pattern transition recognition, whose average recognition accuracy for eight gait pattern transitions reached 99.70%, which increased by 0.15% compared with the highest average recognition accuracy of the single classifier. Moreover, the average recognition accuracy in the absence of different feature data reached 97.47% with good robustness.

## 1. Introduction

Fast, accurate, and stable recognition of gait pattern transition is a prerequisite to the safe and smooth operation of wearable exoskeleton systems. For the practical application of exoskeleton, whether in the military field or civilian field, the exoskeleton system faces a complex and changeable environment. This requires that the gait pattern transition recognition model based on the exoskeleton perception system has good reliability and robustness while ensuring the accuracy. However, there are few studies on gait pattern transition recognition in the field of gait recognition, and the research on the robustness of gait pattern transition recognition model is vacant. How to design a stable and accurate recognition model of gait pattern transition is a highlight in this paper.

With the development of sensor technology and computer technology, multi-source information fusion technology, as an important research direction of intelligent information processing, has been widely used and developed in many fields. The purpose of information fusion is to overcome the shortcomings of a single information source by combining all relevant and available information to obtain a more comprehensive and accurate perception. At present, there is no unified definition of multi-source information fusion due to the extensive and diverse contents of information fusion research. In a general sense, multi-source information fusion technology is a kind of information processing technology that comprehensively processes and utilizes multi-source uncertain information. In addition, there is no research on the application of multi-source information fusion technology in gait pattern transition recognition in the literature we found. Therefore, we will consider the application of multi-source information fusion in gait pattern transition recognition. Different data (information) fusion strategies currently have some impact on the accuracy and stability of gait pattern transition recognition. A good information fusion strategy may improve the accuracy and efficiency of the final recognition, but also improve the anti-interference and fault-tolerant ability of the recognition system [1]. First, data (information) fusion can be divided into data level fusion, feature level fusion, and decision level fusion [2]. The general exoskeleton sensor system includes several types of sensors, but the data level information fusion is only applicable to the same type of sensors, which limits the multi-sensor information fusion at the data level. Data fusion at the decision level is the least dependent on sensors, effectively improving the reliability and robustness of the exoskeleton perception system (gait recognition model), and enhancing the security and stability of the exoskeleton system in complex environments. However, data fusion at the decision level may cause substantial information loss with relatively poor performance. The feature level fusion can give better consideration to the advantages of the data level fusion and the decision level fusion. Such a compromise information fusion can not only keep adequate important information, but also compress the data as much as possible to improve real-time processing. However, it requires a fine preprocessing to the sensor data, including feature extraction and feature selection. Therefore, the hybrid fusion of multi-sensor information at the feature level and decision level is a strong candidate for exoskeleton gait pattern transition recognition.

In addition, according to [3], there were only two ways to improve the performance of the gait pattern transition recognition model: One is to further improve the performance of existing classification algorithms (classifiers); the other is classifier fusion. For the first method, one of the main research directions is to construct a classification model with higher accuracy by improving the classification algorithm or combining the appropriate feature selection algorithm with the classification algorithm [4,5,6,7]. However, there are many classification models for gait pattern recognition [8,9,10,11], and the classification performance is different for different data, and there is no optimal classifier for all problems. The individual performance of the classification method itself also depends on the feature dataset used for classification [12]. The use of multi-classifier fusion (known as classifier ensembles) is now considered a practical and effective solution to solve complex pattern recognition problems and improve classification performance. Different classifiers may provide complementary information about the pattern to be classified, enabling the possibility of better classification performance [13] and less sensitivity to outliers [14]. Literature [14] also points out that classifier fusion does not necessarily improve classification performance, and in some cases, the performance of classifier fusion cannot exceed that of the best single classifier. Therefore, how to select a more suitable multi-classifier model for gait pattern transition recognition is the main priority of this paper. The two key factors that affect the performance of multi-classifier fusion are the diversity of classifier combination and the fusion algorithm [13,14]. This leads to two important questions: (1) How to select classifiers to retain information and achieve diversity in the set of classifiers; (2) how to fuse the classifier outputs to make the final decision [15]. The diversity of multiple classifier models can be achieved by using different classification methods, different numbers and types of features, different training samples, etc. [13]. Much research and applications have been currently carried out on multi-classifier fusion [15,16,17,18,19] to fuse the outputs of these classifiers well, in which D-S evidence theory has been widely studied and applied. In the field of gait and motion pattern recognition, there are also some research and applications related to multiple classifier ensembles [20,21,22]. In this paper, we select a variety of representative classifiers to randomly combine to achieve diversity, and compare and analyze a relatively large number of representative fusion algorithms. Therefore, we study the model that can be used for exoskeleton gait pattern transition recognition in terms of the multi-source information fusion strategy and multi-classifier fusion.

In this paper, a data fusion strategy based on the hybrid fusion of feature level and decision level is proposed for gait pattern transition recognition. First, the optimal feature subset is selected by feature engineering and the corresponding classifier is used for feature level fusion. The classifier selection and fusion algorithm of the multi-classifier fusion model for decision level fusion are then studied, and a relatively optimal multi-classifier fusion model suitable for gait pattern transition recognition is constructed. The focus of this paper is the research on the classifier selection of the multi-classifier fusion model and the multi-classifier fusion algorithm.

## 2. Materials and Methods

To select a better multi-classifier model for gait pattern transition recognition, we selected a relatively suitable decision level fusion method and classifier combination in two stages.

First, we compared the accuracy of multi-classifier models in gait pattern transition recognition under different multi-classifier fusion algorithms. The number of models whose accuracy of the fusion model under different fusion algorithms was higher than or equal to the highest accuracy of a single classifier in the corresponding classifier combination and the number of models with the highest accuracy were counted. According to this, the relatively most effective decision level fusion algorithm and candidate multi-classifier combination were determined.

Second, the perception system of the exoskeleton requires high reliability and robustness in practical applications given the complex actual use environment it faces. To this end, based on the fusion algorithm selected in the first stage, we analyzed the robustness of the multi-classifier model corresponding to the candidate multi-classifier combination under the fusion algorithm, and accordingly selected the suitable classifier combination. In the second stage of the robustness analysis of multi-classifier models, we analyzed the classification performance of each multi-classifier model in the absence of different feature data. Such performance includes the decrease of the accuracy of the multi-classifier model compared with that with the full-feature data; the multi-classifier model with the highest accuracy in the absence of feature data; and the comparison of the average and standard deviation of the accuracy of each multi-classifier model in the absence of several feature data.

**Figure 1:**
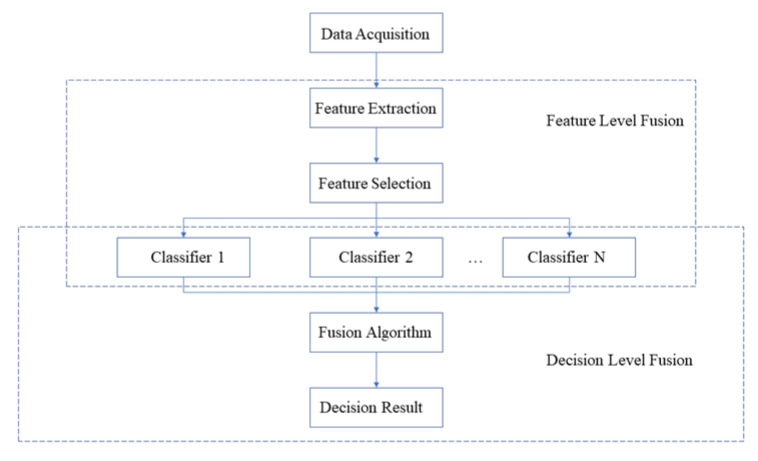
The block diagram of the fusion strategy.

All classification tests in this paper were performed in Matlab (R2019a, The MathWorks, Natick, MA, USA). The block diagram of the fusion strategy is shown in Figure 1.

### 2.1. Data Acquisition and Processing

We collected the data of 22 kinematic parameters (Table 1) of 9 people under 8 gait pattern transitions (Table 2) through a 3D motion capture system with 12 cameras (Motion Analysis, Raptor–4S). The kinematic parameters of both lower limbs were in the sagittal plane, and the parameters of the trunk were in the coronal axis direction in the coronal plane. The 3D motion capture device captured the motion trajectory (coordinate data) of markers on the target object through the cameras, and accurately calculated the body movement information of the target object according to their own algorithm. Before the beginning of the experiment, we corrected the experimental equipment through the calibrator, so as to ensure the accuracy of data acquisition. In addition, we used Helen Hayes whole body model excluding the head for the markers. Specific participants and experimental platforms can be found in our other paper [23]. Participants performed gait transitions as they were accustomed to the behavior. We collected five sets of data of each participant in different transition gait at a sampling frequency of 100 Hz, and preprocessed the data with Cortex software (Butterworth filter, 7 Hz low-pass filtering), and then removed the unsatisfactory data. We finally obtained a data set containing 350 sets of data. In this case, a transition gait starts from the last heel (toe) strike of the trailing limb (limb that enters the new gait pattern second) in the former gait pattern and ends at the first heel (toe) strike of the trailing limb in the next gait pattern. For example, level to up-stair transition was defined as the last heel strike of the trailing limb on the level to the first heel (toe) strike of the trailing limb on the stair.

### 2.2. Feature Extraction and Feature Selection

As mentioned above, in order to achieve better feature level fusion, it is necessary to preprocess the data through feature extraction and feature selection, so as to compress the data as much as possible while keeping enough important information and improve the efficiency of gait pattern transition recognition model. It is also mentioned above that the accuracy of different classification models can be improved by proper feature extraction and feature selection. Therefore, feature extraction and feature selection are key steps in multi-source information fusion.

#### 2.2.1. Feature Extraction

We used Matlab (R2019a, The MathWorks, Natick, MA, USA) to extract features from 22 kinematic parameters of each transition gait in 350 sets of data. About 17 common time domain features (maximum, minimum, peak, peak-to-peak, mean, average amplitude, root amplitude, variance, standard deviation, root mean square, kurtosis, skewness, shape factor, peak factor, pulse factor, margin factor, clearance factor) and 4 common frequency domain features (root mean square of frequency, mean frequency, root variance of frequency, gravity frequency) are extracted from each set of data, totaling to 462 features in time domain and frequency domain. The new dataset obtained after feature extraction contains 350 feature samples as shown in Table 3.

#### 2.2.2. Feature Selection

In this paper, the MRMR (maximum relevance minimum redundancy) –BMSF (binary matrix shuffling filter) second-order feature selection algorithm was used to select the features [24]. First, we screened 100 candidate features that have the maximum correlation with the classes and the minimum redundancy between features from 462 features by MRMR algorithm. Finally, an optimal feature subset containing nine features was selected by BMSF algorithm. The feature subset is shown in Table 4.

Of these, MRMR is a feature selection algorithm or criterion proposed by Peng et al. [25], which uses mutual information to measure the correlation between features and classes and the redundancy between features and features, and selects the feature that has the greatest correlation with classes and the least redundancy with selected features; BMSF is a data-driven heuristic random search algorithm proposed by Zhang et al. [26,27], implemented by combining binary matrix shuffling with SVM to perform filtering. Although BMSF is a wrapper feature selection algorithm, its generalization degree is high, and its advantages are not limited to support vector machine (SVM) classifier. Since this paper focused on the research of multi-classifier fusion model for gait pattern transition recognition, the MRMR and BMSF algorithms would not be described in detail here.

In order to analyze the classification performance of multiple classifier models in the absence of different feature data, we regarded the relevant time domain and frequency domain features of the same kinematic parameter as a type. The feature subset can be divided into five types of feature data: feature data related to right hip angular velocity, feature data related to right thigh velocity, feature data related to left hip angular velocity, feature data related to left shank velocity, and feature data related to left ankle angular velocity. Then we analyzed the classification performance of multi-classifier model in the absence of a certain type of feature data. This would simulate the reliability and robustness of the exoskeleton in the case of a certain type of sensor failure in practical applications.

### 2.3. Multi-Classifier Fusion

#### 2.3.1. Fusion Algorithm

As mentioned above, there are much research on multi-classifier fusion algorithms, and they are also applied in the field of gait and motion recognition. Here, we selected representative related fusion algorithms for comparative analysis.

1. D-S theory

D-S evidence theory is a typical reasoning method for intelligent processing and data fusion of uncertain information. It has been widely used in various fields [28,29,30,31,32]. Under the D-S evidence theory, a complete set of mutually incompatible basic propositions (presumptions) is called a recognition frame, which represents all possible answers to a question with only one correct answer. The subset of this framework is called a proposition. The degree of confidence assigned to each proposition is called the basic probability assignment (BPA, also known as the m function), and *m* (*A*) is the basic probability assignment, which reflects the degree of confidence in A. The belief function Bel (A) represents the exact degree of belief in the proposition A, and the likelihood function Pl (A) represents the degree of belief that the proposition A is not false, that is, the measure of uncertainty that it seems possible for A to be true. In fact, [Bel (A), Pl (A)] represents the uncertainty interval of A, and [0, Bel (a)] represents the supporting evidence interval of proposition A. [0, Pl (A)] denotes the quasi-belief interval of proposition A, [Pl (A), 1] denotes the rejection evidence interval of proposition A. Let *m*_1_ and *m*_2_ be the basic probability assignment functions derived from two independent evidence sources, then Dempster’s combination rule can be used to calculate the new basic probability assignment function. Such function is produced by the interaction of the two evidences and reflects the fusion information. Dempster’s combination rule is as follows:

(1) $$m\left(\emptyset \right)=0;\;m\left(A\right)=\frac{\sum_{A_{1}\cap A_{2}=A}m_{1}\left(A_{1}\right)m_{2}\left(A_{2}\right)}{\sum_{A_{1}\cap A_{2}\neq \emptyset }m_{1}\left(A_{1}\right)m_{2}\left(A_{2}\right)},\;A\neq \emptyset$$

2. Majority voting

The basic idea of the voting method is “the minority is subordinate to the majority”, where all the member classifiers participate in the voting, the candidates are all the possible classification results, and the classification result with the most votes is the final output result. In this method, each member classifier is regarded as a completely equal individual, and all member classifiers are regarded as a voter. The majority voting method is a voting method, which requires more than half of valid votes.

3. Decision Templates

Decision templates for multiple classifier fusion was proposed by Kuncheva in 1999 [16]. First, the DT method establishes the output matrix of the classifier in the fusion system and a DT matrix for each class; then the two are compared and the similarity is calculated. The greater the similarity is, the greater the support for the class is. Finally, the class corresponding to the highest similarity is selected as the judgment result.

In order to describe the following basic elementary fusion algorithm more easily, we assume that there are *n* classifiers X = (x_1_, x_2_, …, x*_n_*), *m* classes Y = (y_1_, y_2_, …, y*_m_*). z*_ij_* represents the probability (score) of the sample to be recognized as the *j* class by classifier x*_i_*, *i* = 1, 2, …, *n*; *j* = 1, 2, …, *m*. After n classifiers are fused, a vector *e_j_* is obtained, which represents the overall likelihood that the sample to be identified belongs to each class. Finally, the class corresponding to the maximum value of *e_j_* is selected as the input pattern.

4. Sum

The scores provided by each base classifier are summed and the class label with the highest score is assigned to a given input pattern.

(2) $$e_{j}=\overset{n}{\underset{i=1}{\sum }}z_{ij},\;j=1,2,\ldots ,m$$

5. Average

Find the average of the scores for each class between the classifiers and assign the input pattern to the class with the highest score in the average. It is equivalent to the sum of the rules.

(3) $$e_{j}=\frac{1}{n}\overset{n}{\underset{i=1}{\sum }}z_{ij},\;j=1,2,\ldots ,m$$

6. Maximum

Find the maximum score for each class between the classifiers and assign the input pattern to the class with the highest score among the maximum scores.

(4) $$e_{j}=\max_{i=1}^{n}\left\{z_{ij}\right\},\;j=1,2,\ldots ,m$$

7. Minimum

Find the minimum of each class between the classifiers and assign the input pattern to the class with the highest score among the minimum scores.

(5) $$e_{j}=\min_{i=1}^{n}\left\{z_{ij}\right\},\;j=1,2,\ldots ,m$$

8. Product

The scores provided by each base classifier are multiplied and the class label with the highest score is assigned to a given input pattern.

(6) $$e_{j}=\overset{n}{\underset{i=1}{\prod }}z_{ij},\;j=1,2,\ldots ,m$$

Due to space limitations, the specific theory of each fusion algorithm is not introduced in detail in this paper.

#### 2.3.2. Selection of Classifiers

There are many classifiers for gait recognition [8,10,11,33], such as support vector machines (SVM) [10], K-nearest neighbor (KNN), neural networks [4], Bayesian classifiers [8], etc.

We selected five commonly used representative classifiers as the candidate base classifiers for the multi-classifier model of gait pattern transition recognition. The SVM (linear, RBF, polynomial) with three different kernel functions, KNN (K = 1) and BP (one hidden layer, 10 neurons, and “tansig” activation function) are included. In order to analyze the multi-classifier model more conveniently, we numbered the five classifiers (as shown in Table 5), and replaced each classifier with a number for description hereinafter.

### 2.4. Evaluation Method

After reviewing relevant literatures where most of them take the accuracy as the evaluation criterion of the performance of multi-classifier model, this paper also took the accuracy as the evaluation criterion of the performance of the whole multi-classifier model. The recognition accuracies of each multiple classifier model and the single classifier corresponding to each multi-classifier model under different fusion algorithms were obtained through a ten-fold cross-validation method, and the relevant accuracies in the absence of different feature data were obtained in the same way. The above process was repeated 100 times, and the relevant average accuracy of each multi-classifier model was finally obtained.

## 3. Results

For better comparative analysis, in the first stage, we did not list all the relevant single classifier accuracy and fusion accuracy in all multi-classifier models. Only the accuracies of the multi-classifier models whose accuracy was higher than or equal to the highest accuracy in the single classifier and the highest accuracies of the related single classifier were listed as shown in Table 6. The italics in the table represents the multi-classifier fusion model whose accuracy was higher than or equal to the highest accuracy of the relevant single classifier; and the bold indicated the multi-classifier fusion model with the highest accuracy for each multi-classifier combination.

In the second stage, according to the selected fusion algorithm, we analyzed the classification performance of the correlation multi-classifier fusion model under the fusion algorithm in five different cases of missing feature data. Similarly, we also only listed the accuracies of the related multiple classifier fusion models and the highest accuracies of a single classifier in these models, as shown in Table 7. In order to better analyze the advantages and disadvantages of the multi-classifier fusion model, we listed the fusion accuracy and their standard deviation of the relevant multi-classifier fusion model in Table 8, where the bold indicated the highest accuracy of the multi-classifier fusion model in each case of missing feature data.

## 4. Discussion

First, we can see from the above results (Table 6) that there were more multi-classifier models whose accuracy was higher than or equal to the accuracy of single classifier under the D-S algorithm. Moreover, the highest accuracy mostly appeared in the model based on the D-S algorithm among these multi-classifier models. Therefore, we adopted the D-S algorithm as the decision level fusion algorithm of the multi-classifier model of gait pattern transition recognition, and carried out subsequent research accordingly.

Moreover, in order to select a better classifier combination, we further compared and analyzed the robustness of the multi-classifier model whose accuracy was higher than the highest accuracy of the single classifier under the D-S algorithm. We found an insignificant decrease in the classification accuracy of most multi-classifier models in the absence of a variety of different feature data when compared with the classification accuracy under full-feature data (as shown in Table 6 and Table 7). In addition, the accuracy of fusion decision in almost all multi-classifier models was still higher than the highest accuracy of a single classifier. This illustrated the stability of the D-S algorithm in these multi-classifier models and its low sensitivity to anomalies. That is to say, in the practical application of exoskeleton, when some sensors of the perception system fail or data are missing, the gait pattern transition recognition system can still maintain good robustness and reliability. Additionally, in the case of missing five types of feature data, the highest accuracy occurred twice in the combination of 3 and 4 classifiers and the combination of 3, 4, and 5 classifiers, respectively.

Finally, we can see from Table 8 that in the case of missing different feature data, the multi-classifier combination model with the highest average accuracy was the combination of 3, 4, and 5 (97.47%), and the standard deviation (0.0216) was not the smallest, but only higher than the combination of 1 and 4 (0.0174). On the whole, the combination of 3, 4, and 5 was thus a multi-classifier combination model which was relatively most suitable for gait pattern transition recognition. It can be seen from Table 6 that the average recognition accuracy of the model for eight gait pattern transitions reached 99.70%, which was 0.15% higher than the highest average recognition accuracy of the single classifier (99.55%). In addition, the literature [34] used relevant kinematic data to compare various latest feature processing and dimensionality reduction methods and machine learning classifiers to find an effective tool for recognition of LMTS (locomotion mode transitions). They finally found that the average recognition accuracy of all machine learning classifiers for the LMTS was 99.6%, while the recognition accuracy of the multi-source in-formation fusion model we selected was 99.7%, which was consistent with the conclusion mentioned in this paper that the average recognition accuracy of this model for eight gait pattern transitions is increased by 0.15% compared with the highest average recognition accuracy of a single classifier. It also proved the effectiveness of multi-source information fusion in improving the accuracy of gait pattern transition recognition.

It should be noted that we do not consider the factor of recognition efficiency in the selection of multi-classifier model. In terms of the efficiency of the multi-classifier model, under the same circumstances, the fewer the classifiers are, the lower the computational complexity of the classifier is, and the higher the efficiency of the multi-classifier model is. However, the recognition efficiency of different classifiers varies with respect to the gait pattern transition recognition. Therefore, the efficiency of the final multi-classifier model also needs to integrate the number of classifiers in the multi-classifier model and the recognition efficiency of each classifier; it is undergoing further study. In future work, we will consider using IMU equipment to re-collect data and verify the effectiveness of the multi-source information fusion model constructed in this paper as a supplement to this paper.

## 5. Conclusions

In this paper, we studied the application of multi-source information fusion technology in exoskeleton gait pattern transition recognition from two aspects: multi-source information fusion strategy and multi-classifier fusion. A hybrid fusion strategy based on feature level data fusion and decision level data fusion was proposed for exoskeleton gait pattern transition recognition. The multi-classifier model of data fusion in decision level was analyzed in terms of two aspects of classifier selection and multi-classifier fusion algorithm. Finally, a multi-classifier model based on D-S evidence theory and the combination of three SVM classifiers with different kernel functions (linear, RBF, polynomial) was constructed, which had higher gait pattern transition recognition accuracy and robustness. This multi-source information fusion model can not only improve the accuracy of gait pattern transition recognition, but also improve the anti-interference and fault tolerance of gait pattern transition recognition system of exoskeleton through the multi-source information hybrid fusion strategy of feature level and decision level. Finally, we also verified the robustness of the model by analyzing the performance of the model in the case of missing feature data.

## Figures and Tables

**Table 1:** Abbreviation and description of kinematic parameters.

No.	Abbr.	Description
1	rt aa	right hip angular acceleration
2	lt aa	left hip angular acceleration
3	rs aa	right knee angular acceleration
4	ls aa	left knee angular acceleration
5	rf aa	right ankle angular acceleration
6	lf aa	left hip angular acceleration
7	rt av	right hip angular velocity
8	lt av	left hip angular velocity
9	rs av	right knee angular velocity
10	ls av	left knee angular velocity
11	rf av	right ankle angular velocity
12	lf av	left ankle angular velocity
13	ls v	left shank velocity
14	lt v	left thigh velocity
15	ls a	left shank acceleration
16	lt a	left thigh acceleration
17	rt a	right thigh acceleration
18	rs a	right shank acceleration
19	rt v	right thigh velocity
20	rs v	right shank velocity
21	trunk a	trunk acceleration
22	trunk v	trunk velocity

**Table 2:** Abbreviation and description of gait pattern transitions.

No.	Abbr.	Description
1	L-R UP	level walking to up-ramp walking transition
2	R-L DOWN	down-ramp walking to level walking transition
3	L-S UP	level walking to up-stair walking transition
4	S-L DOWN	down-stair walking to level walking transition
5	R-L UP	up-ramp walking to level walking transition
6	L-R DOWN	level walking to down-ramp walking transition
7	S-L UP	up-stair walking to level walking transition
8	L-S DOWN	level walking to down-stair walking transition

**Table 3:** New dataset.

Gait Transitions	Samples	Features
L-R UP	45	462
R-L DOWN	45	462
L-S UP	44	462
S-L DOWN	42	462
R-L UP	44	462
L-R DOWN	43	462
S-L UP	44	462
L-S DOWN	43	462

**Table 4:** The features of feature subset.

Features
rt av Mean; lt av Mean; lt av Skewness; lf av Mean; lf av Kurtosis; lf av Skewness; ls v Shape Factor; rt v Peak–To–Peak; lt av Root Variance of Frequency

**Table 5:** The candidate base classifiers.

No.	Classifiers
1	BP (one hidden layer, 10 neurons, and “tansig” activation function)
2	KNN (K = 1)
3	SVM (linear)
4	SVM (RBF)
5	SVM (polynomial)

**Table 6:** The accuracies of the related multiple classifier fusion models (%).

Classifier Ensemble	Fusion Algorithm								
	Single Max	Majority Voting	Maximum	Sum	Minimum	Average	Product	Decision Template	D-S
1, 2	96.09	94.17	94.34	***96.09***	95.99	***96.09***	95.99	96.05	***96.09***
1, 4	94.60	93.23	*94.60*	*96.43*	*94.69*	*96.43*	***97.45***	*96.91*	***97.45***
2, 4	96.32	93.63	***96.32***	***96.32***	***96.32***	***96.32***	***96.32***	***96.32***	***96.32***
3, 4	99.78	96.17	*99.83*	***99.87***	98.39	***99.87***	99.74	***99.87***	***99.87***
3, 5	99.74	99.70	*99.83*	***99.87***	***99.87***	***99.87***	*99.83*	***99.87***	***99.87***
1, 2, 4	95.99	***96.83***	94.66	*96.77*	95.90	*96.77*	95.90	*96.60*	*96.73*
1, 3, 5	99.65	***99.70***	95.34	99.37	99.35	99.37	99.35	99.22	99.59
3, 4, 5	99.55	*99.61*	***99.70***	***99.70***	98.81	***99.70***	***99.70***	***99.70***	***99.70***
1, 3, 4, 5	99.74	*99.74*	95.02	99.72	98.88	99.72	99.66	99.65	***99.80***

The italics in the table indicated the multi-classifier fusion model whose accuracy was higher than or equal to the highest accuracy of the relevant single classifier; The bold indicated the multi-classifier fusion model with the highest accuracy for each multi-classifier combination.

**Table 7:** The accuracies (%) of the related multiple classifier fusion models.

Classifier Ensemble	No rt av Features		No rt v Features		No lt av Features		No ls v Features		No lf av Features	
	Single Max	D-S Ensemble	Single Max	D-S Ensemble	Single Max	D-S Ensemble	Single Max	D-S Ensemble	Single Max	D-S Ensemble
1, 2	95.09	95.09	94.40	92.84	92.73	92.73	96.09	96.09	89.41	89.41
1, 4	93.63	96.55	94.79	96.50	93.59	94.24	94.67	97.27	90.14	92.60
2, 4	95.04	95.04	92.39	92.39	93.07	92.67	96.12	96.12	89.63	89.51
3, 4	98.39	98.96	98.85	98.55	93.62	**95.84**	99.46	99.65	90.24	93.89
3, 5	98.46	**99.20**	99.13	**99.11**	92.38	92.42	99.48	99.48	91.64	92.07
3, 4, 5	98.40	98.94	99.39	98.83	93.58	95.81	99.39	**99.70**	91.82	**94.05**
1, 3, 4, 5	98.40	99.15	99.20	98.75	93.89	95.40	99.78	99.09	91.50	93.61

The bold in the table indicated the highest accuracy of the multi-classifier fusion model in each case of missing feature data.

**Table 8:** The accuracies (%) and standard deviations of the related multiple classifier fusion models.

Classifier Ensemble	No rt av Features	No rt v Features	No lt av Features	No ls v Features	No lf av Features	Average	Standard Deviation
1, 2	95.09	92.84	92.73	96.09	89.41	93.23	0.0231
1, 4	96.55	96.50	94.24	97.27	92.60	95.43	0.0174
2, 4	95.04	92.39	92.67	96.12	89.51	93.15	0.0230
3, 4	98.96	98.55	**95.84**	99.65	93.89	97.38	0.0217
3, 5	**99.20**	**99.11**	92.42	99.48	92.07	96.46	0.0344
3, 4, 5	98.94	98.83	95.81	**99.70**	**94.05**	**97.47**	0.0216
1, 3, 4, 5	99.15	98.75	95.40	99.09	93.61	97.20	0.0228

The bold in the table indicated the highest accuracy of the multi-classifier fusion model in each case of missing feature data.

## Data Availability

The data presented in this study are available on request from the corresponding author. The data are not publicly available due to the data also forms part of an ongoing study.
